# Identification of a necroptosis-related gene signature as a novel prognostic biomarker of cholangiocarcinoma

**DOI:** 10.3389/fimmu.2023.1118816

**Published:** 2023-03-02

**Authors:** Lixia Xu, Xueping Gao, Jiyuan Xing, Zhixian Guo

**Affiliations:** ^1^Department of Infectious Diseases, The First Affiliated Hospital of Zhengzhou University, Zhengzhou, Henan, China; ^2^Department of Clinical Laboratory Medicine, Southwest Hospital, Third Military Medical University (Army Medical University), Gaotanyan, Chongqing, China

**Keywords:** necroptosis, cholangiocarcinoma, prognostic signature, immune microenvironment, biomarker

## Abstract

**Background:**

Cholangiocarcinoma (CHOL) is the most prevalent type of malignancy and the second most common form of primary liver cancer, resulting in high rates of morbidity and mortality. Necroptosis is a type of regulated cell death that appears to be involved in the regulation of several aspects of cancer biology, including tumorigenesis, metastasis, and cancer immunity. This study aimed to construct a necroptosis-related gene (NRG) signature to investigate the prognosis of CHOL patients using an integrated bioinformatics analysis.

**Methods:**

CHOL patient data were acquired from the Gene Expression Omnibus (GEO) (GSE89748, GSE107943) and The Cancer Genome Atlas (TCGA) databases, with NRGs data from the necroptosis pathway in the Kyoto Encyclopedia of Genes and Genomes (KEGG) database. Univariate and multivariate regression analyses were performed to establish the NRG signatures. Kaplan–Meier (KM) curves were used to evaluate the prognosis of patients with CHOL. Functional enrichment analysis was performed to identify key NRG-associated biological signaling pathways. We also applied integrative multi-omics analysis to the high- and low-risk score groups. Spearman’s rank correlation was used to clarify the relationship between the NRG signature and immune infiltration.

**Results:**

65 differentially expressed (DE) NRGs were screened, five of which were selected to establish the prognostic signature of NRG_S_ based on multivariate Cox regression analysis. We observed that low-risk patients survived significantly longer than high-risk patients. We found that patients with high-risk scores experienced higher immune cell infiltration, drug resistance, and more somatic mutations than patients with low-risk scores. We further found that sensitivities to GW843682X, mitomycin C, rapamycin, and S-trityl-L-cysteine were significantly higher in the low-risk group than in the high-risk group. Finally, we validated the expression of five NRGs in CHOL tissues using the TCGA database, HPA database and our clinical data.

**Conclusion:**

These findings demonstrate that the five-NRG prognostic signature for CHOL patients is reasonably accurate and valid, and it may prove to be of considerable value for the treatment and prognosis of CHOL patients in the future.

## 1 Introduction

Cholangiocarcinoma (CHOL) is a highly heterogeneous malignancy stemming from biliary epithelia. CHOL is the most prevalent type of malignancy and the second most common form of primary liver cancer, accounting for approximately 20% of all primary liver cancers (1, 2). Surgical treatment, immunotherapy, chemotherapy, and other comprehensive tumor treatment methods have changed the prognosis of many patients with CHOL. Patients with CHOL nonetheless still tend to have unfavorable prognoses, with only 10% of patients surviving for five years (3). The main factors contributing to poor prognosis are the heterogeneity, infiltrative nature, and rapid drug resistance of CHOL, making it difficult to completely remove the tumor by surgical procedures and identify the therapeutic target of CHOL (1, 4, 5). There is, therefore, a pressing need to further explore the occurrence and progression of CHOL to improve the treatment and survival rates of CHOL patients.

Necroptosis is a self-destruction cellular process that is regulated *via* a complex signaling cascade (6), and it is closely related to key aspects of cancer biology regulation, including tumorigenesis, metastasis, and cancer immunity (7, 8). There is increasing evidence that overcoming apoptosis resistance by induction of cancer cell necroptosis may be an attractive therapeutic approach for patients with CHOL (9–11). For instance, the application of both TNFα and gemcitabine has been shown to induce RIPK1/RIPK3/MLKL-dependent necrosis when apoptosis-inhibitory proteins and caspases are blocked, as evidenced by increased expression of RIPK3 and MLKL in CHOL cell lines (9, 12). In addition, Xu et al. found that the alkaloid matrine can induce necroptosis in CHOL by enhancing the expression of RIP3 and the RIP3/MLKL/ROS signaling pathway, thus providing a new individualized strategy for overcoming chemoresistance in CHOL therapy based on the expression of RIP3 (12). Hence, exploring the role of necroptosis in tumorigenesis and the progression of CHOL has great potential for the diagnosis and treatment of CHOL patients. The rapid development of high-throughput sequencing and multi-omics studies has allowed a substantial body of reliable information to be obtained regarding the treatment and prognosis of patients with CHOL (13–15).

In this study, we first profiled the necroptosis-related genes in CHOL and developed a risk prediction model based on five genes to explore their functional enrichment and ability to predict outcomes. The performance of the prediction models was validated in three independent cohorts (TCGA, GSE89748, and GSE107943). Additionally, we examined the differences in drug resistance, somatic mutations, and immune infiltration between the low- and high-risk groups. In brief, our prognostic signature provides a reliable method for predicting the prognosis of patients with CHOL, and it offers clinicians a reference for early diagnosis and treatment of CHOL.

## 2 Materials and methods

### 2.1 Data collection and preprocessing

TCGA biolinks was used to extract RNA-Seq data from 36 CHOL and 9 normal samples, as well as relevant clinical information from TCGA database (http://portal.gdc.cancer.gov) (16). Additionally, the University of California Santa Cruz (UCSC) provided FPKM, somatic mutation, and clinical data on CHOL. In the present study, CHOL datasets GSE89748 and GSE107943 (17, 18) from the GEO database (https://www.ncbi.nlm.nih.gov/geo) were downloaded using the GEO query R package, which was used as the external validation set, including available expression profile data and clinical information of bile duct cancer samples. In total, 72 CHOL samples from the GSE89748 dataset and 30 CHOL samples from the GSE107943 dataset were acquired. A total of 159 necroptosis-associated genes (NRGs) were obtained from the necroptosis pathway (hsa04217) in the Kyoto Encyclopedia of Genes and Genomes (KEGG) database.

### 2.2 Identification of the expression patterns and biological functions of DENRGs in CHOL

First, we extracted the NRG_S_ expression matrix from TCGA and then screened for differentially expressed necroptosis-related genes (DENRGs) between the CHOL and normal groups using the limma package (19). Significant DENRGs were visualized using volcano plots constructed using the ggplot2 package. The criteria for differentially expressed genes (DEGs) were FDR < 0.05 and |log_2_FC| > 1. Furthermore, differences in DENRGs between the CHOL and normal groups were visualized using boxplots. DENRGs were also analyzed based on a protein-protein interaction (PPI) network using the STRING database (20), and correlations between them were visualized using heatmaps. To investigate the biological role of DENRGs, we examined biological processes (BP), cellular components (CC), and molecular functions (MF) according to the Gene Ontology (GO) database and KEGG signaling pathways using the R tool cluster Profile (21). The enrichment significance thresholds were set at an adjusted p-value of < 0.05.

### 2.3 Development and validation of DENRGs-based prognostic models

DENRGs were first identified for their prognostic values in the TCGA cohort by univariate Cox proportional hazards regression analysis, and the genes with p*-*values < 0.05 were then entered into the multivariate Cox regression analysis. A risk score model was built based on the expression levels of the prognosis-associated genes and the contribution coefficient (and beta) of the multivariate Cox proportional hazard regression model. Based on the above risk score model, we calculated the prognostic risk value for each patient sample in TCGA (training cohort), GSE89748 (validation cohort 1), and GSE107943 (validation cohort 2). All CHOL samples were divided into high- and low-risk groups, with the median risk score as the cutoff value. Kaplan–Meier survival analyses were performed using the ‘survival’ and ‘survminer’ (22) packages between the high- and low-risk groups. To further assess the clinical diagnostic value of the risk score, time-dependent receiver operating characteristic (ROC) curves for overall survival (OS) and area under the ROC curves (AUCs) at 1, 3, and 5 years in TCGA (training cohort), GSE89748 (validation cohort 1), and GSE107943 (validation cohort 2) were generated using the R package “survivalROC” (23). OS is defined as the time from randomization to death. Furthermore, we constructed a risk plot to explore the relationship between the risk score and the prognosis status.

### 2.4 Process of the screening signature for the Cox regression model and building of the nomogram models

Univariate Cox regression was performed to examine the relationship between patient clinical characteristics (age, sex, stage, pathology, weight, height, and BMI), risk score, and OS. Significant prognostic factors (p < 0.05) in the univariate analyses were selected for multivariate Cox regression analysis. Forest plots were used to present the results of the univariate and multivariate Cox analyses, including all of the above variables. A nomogram was built based on the identified variables in the multivariate Cox regression analysis to facilitate clinical application.

### 2.5 Exploration of differences in biological functions between CHOL subgroups

To determine the differences in biological functions between the high- and low-risk groups, DEGs between the two groups were screened using the limma R package with FDR thresholds of < 0.05, and absolute log_2_FC > 1. A volcano plot was then used to illustrate the DEGs using ggplot2. To visualize the expression patterns of DEGs between the low- and high-risk groups, we used R package (pheatmap) to generate a heatmap. All DEGs were subjected to GO and KEGG pathway enrichment analyses using Metascape (http://metascape.org) (24). A p-value < 0.01 and a minimum of three counts were set as the cutoff criteria for selecting significant enrichment results. GO and KEGG analyses were also performed using the R package “cluster Profiler” to explore the underlying biological roles of the DEGs (21). The enrichment results were visualized using bar and dot plots. Gene set enrichment analysis (GSEA) (25) was performed using cluster Profiler, with a p-value of < 0.05 as the threshold for significantly enriched KEGG pathways. The top 20 significantly enriched pathways ranked by normalized enrichment scores were visualized using a ridgeline plot.

### 2.6 Applying integrative multi-omics analysis between the high- and low-risk score groups

The R package “Rcircos” (26) was used to map the chromosomal locations of clinically significant NRGs. The Friends tool was then used to functionally annotate these genes, which were subsequently estimated by semantic analysis using the R package GOSemSim (27). By building a ridgeline regression model based on the Genomics of Drug Sensitivity in Cancer (GDSC) database (www.cancerrxgene.org/), we predicted the half-maximal inhibitory concentration (IC_50_) for chemotherapy drugs in the high- and low-risk groups and we inferred the sensitivity of the patients (28). To detect somatic mutations in CHOL patients between the high-risk and low-risk subgroups, we used the mutation annotation format (MAF) in TCGA database. The results were visualized using a waterfall plot (oncoplot). Using the online tool Network Analyst (29), we explored the transcriptional regulators and chemical targets of hub necroptosis genes based on the JASPAR Tarbase and mir-Tarbase databases.

### 2.7 Correlation analysis between the prognostic DENRGs and immune cell infiltration

Immune infiltration is a significant factor in tumor progression, treatment, and prognosis. We used the “ESTIMATE” R package to estimate the stromal score, immune score, and tumor purity in the high- and low-risk subgroups (30). The R package “ggplot2” was then applied to generate boxplots to visualize differences between the two groups for the above-mentioned immune scores and tumor purity. CIBERSORT is a deconvolution algorithm that can calculate the infiltration abundance of 22 immune cell types in all tumor samples (31). Heatmaps were drawn using the R package pheatmap to illustrate the fractions of immune cell types for each sample, and a correlation analysis between 22 immune cell types and prognostic necroptosis genes was performed using the corrplot package. The results were visualized using the ‘pheatmap’ package. Immune infiltration differences between the high- and low-risk groups of CHOL patients were determined using the ggplot2 package. Additionally, the most positively and negatively correlated gene-immune cell pairs were displayed using a scatter plot.

### 2.8 Immunohistochemical analysis of five NRGs in HPA

The protein expression of the five NRGs between CHOL and normal tissues was measured by immunohistochemistry from the Human Protein Atlas (HPA) (https://www.proteinatlas.org/), which is a valuable database providing the data of immunohistochemistry expression for specific human tissues and cells (32).

### 2.9 Tumor samples collection and qRT-PCR

A total of 12 CHOL tissue samples and 10 corresponding normal hepatobiliary duct tissues were obtained from patients who underwent surgical resection between March 2021 and October 2022 at the First Affiliated Hospital of Zhengzhou University, Henan, China. The samples were immediately frozen in liquid nitrogen after tissue resection. The total RNA of the tissue samples was extracted using TRIzol reagent (Invitrogen) according to the manufacturer’s protocol. The RNA samples were reverse-transcribed into cDNA by using iScriptTM cDNA Synthesis Kit. RT-qPCR was performed using a thermal cycler (Roche LightCycler 480) using IQTM SYBR^®^ Green Supermixes for Real-Time PCR. The mRNA expression was normalized to the expression of glyceraldehyde-3-phosphate dehydrogenase (GAPDH) mRNA and counted by the 2−ΔΔCt method. The PCR primer sequences are shown in **Table 1**. This study conforms to the guidelines issued in the Declaration of Helsinki and was approved by the Ethics Committee of the First Affiliated Hospital of Zhengzhou University (Approval Number: SS-2019-018).

**Table 1:** Primer list of PCR.

Gene Name	Forward primer	Reverse primer
GAPDH	GGAGCGAGATCCCTCCAAAAT	GGCTGTTGTCATACTTCTCATGG
PYGB	AGGTGCGGAAGAGCTTCAAC	TCGCGCTCGTAGTAGTGCT
IFNGR2	CTCCTCAGCACCCGAAGATTC	GCCGTGAACCATTTACTGTCG
TICAM1	GCCAGCAACTTGGAAATCAGC	GGGGTCGTCACAGAGCTTG
STAT6	GTTCCGCCACTTGCCAATG	TGGATCTCCCCTACTCGGTG
VPS4B	ATGTCATCCACTTCGCCCAAC	TTGCTTGGCTTTATCACCCTG

### 2.10 Statistical analysis

All data processing and statistical analyses were performed using R software (version 4.2.1). A detailed description of the bioinformatics analyses is provided in the corresponding subsections. * p < 0.05; ** p < 0.01; *** p < 0.001. *A* p-value < 0.05 was taken as representing statistical significance.

## 3 Results

### 3.1 Identification of DENRGs

**Figure 1:**
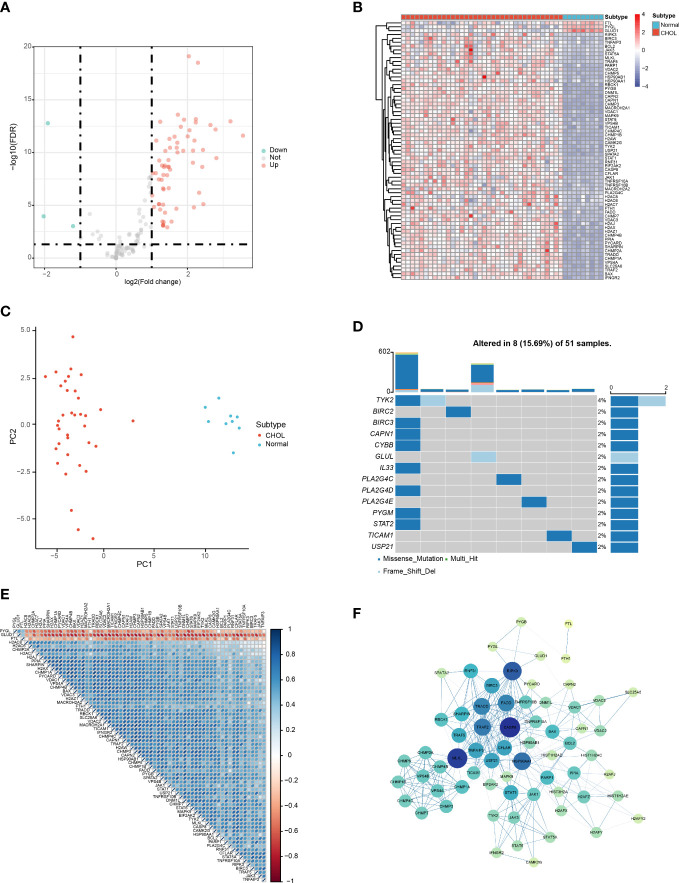
Identification of DENRGs in the CHOL group. **(A)** Volcano plot of the DENRGs. Genes indicated in red, blue, and gray colors were significantly upregulated (Up), downregulated (Down), or not significantly different (Not), respectively. **(B)** Heatmap showing the expression of 65 DENRGs in the normal and CHOL samples. Red, CHOL group; Blue, normal group **(C)** Principal components analysis (PCA) indicating the expression patterns of DENRGs. **(D)** Oncoplot of the DENRG mutations. **(E)** Heat map of the correlation between the DENRGs. Red colors indicate positive correlations and blue colors represent negative correlations. The darker the color, the stronger the correlation. **(F)** PPI network of the DENRGs. The larger the node, the higher the number of interactions with other genes, and the thicker the line, the higher the correlation coefficient.

According to the filter criteria, a total of 67 DENRGs were screened, including 64 upregulated genes and 3 downregulated genes. The expression distribution of the DENRGs was visualized using volcano plots (**Figure 1A**). Based on the boxplot and heatmap, it was clear that *H2AW, PYGB, PYCARD, CAPN2, BIRC3, H2AX, CHMP4C, STAT1, CHMP3, CHMP4B, CAPN1, H2AZ1*, and *BAX* were highly expressed in the CHOL group, whereas *FTL*, *GLUD1*, and *PYGL* were expressed at very low levels compared with the normal group (**Figure S1**; **Figure 1B**). Principal component analysis (PCA) of these DENRGs clearly distinguished the CHOL group from the control group (**Figure 1C**). Mutation analysis indicated that missense mutations were the most common, and *TYK2* had the highest mutation rate, which was a missense mutation with a frameshift deletion (**Figure 1D**). The heat map showed that *FTL*, *GLUD1*, and *PYGL* were positively correlated with each other and negatively correlated with the other DENRGs (**Figure 1E**). Furthermore, the PPI network diagram suggested that *CASP8*, *MLKL*, and *RIPK3* exhibited the strongest interactions with the other DENRGs (**Figure 1F**).

### 3.2 GO and KEGG functional analysis of the DENRGs

**Figure 2:**
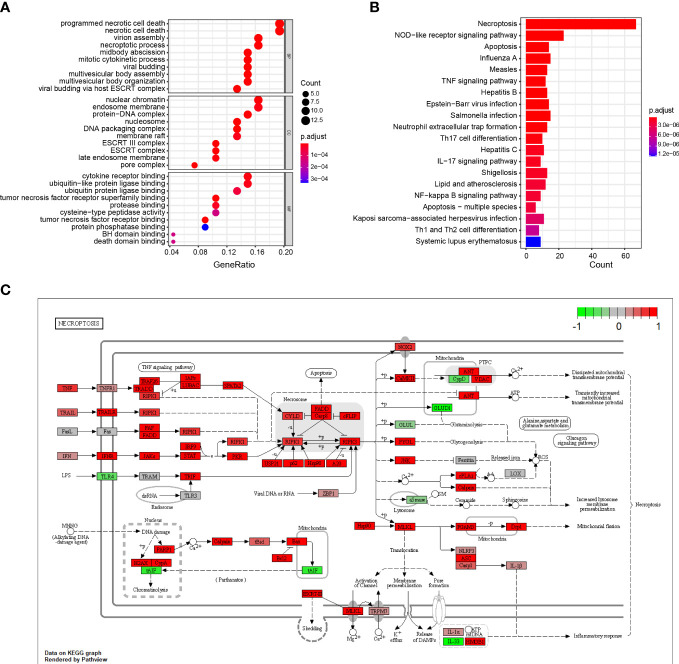
GO and KEGG enrichment analysis of DENRGs. **(A)** Dot plot showing the top 10 biological functions enriched in Gene Ontology (GO) terms. **(B)** Bar plot showing the top 10 signaling pathways enriched in KEGG terms. **(C)** Diagrammatic outline of the necroptosis pathway.

The results show that the DENGs were mainly related to cell death processes, such as programmed necrotic cell death, midbody abscission, necrotic cell death, mitotic cytokinetic process, necrotic process and virtual budding, and ESCRT complex, nucleosome, DNA packaging complex, protein DNA complex, nuclear chromatin, tumor necrotic factor receiver superfamily binding, tumor necrotic factor receiver binding, cytokine receiver binding, ubiquitin-like protein ligase binding, and protein binding (**Figure 2A**; **Table S1**). The KEGG results suggest that the DENRGs were mainly involved in multiple functional pathways (e.g., Necroptosis, NOD-like receptor signaling pathway, Apoptosis, Influenza A, TNF signaling pathway, Th17 cell differentiation, IL-17 signaling pathway, and Neutrophil extracellular trap formation pathway) (**Figure 2B**; **Table S1**). A panoramic view of the necroptosis pathway in KEGG was generated (**Figure 2C**).

### 3.3 Construction of a prognostic model within necroptosis-associated genes

**Figure 3:**
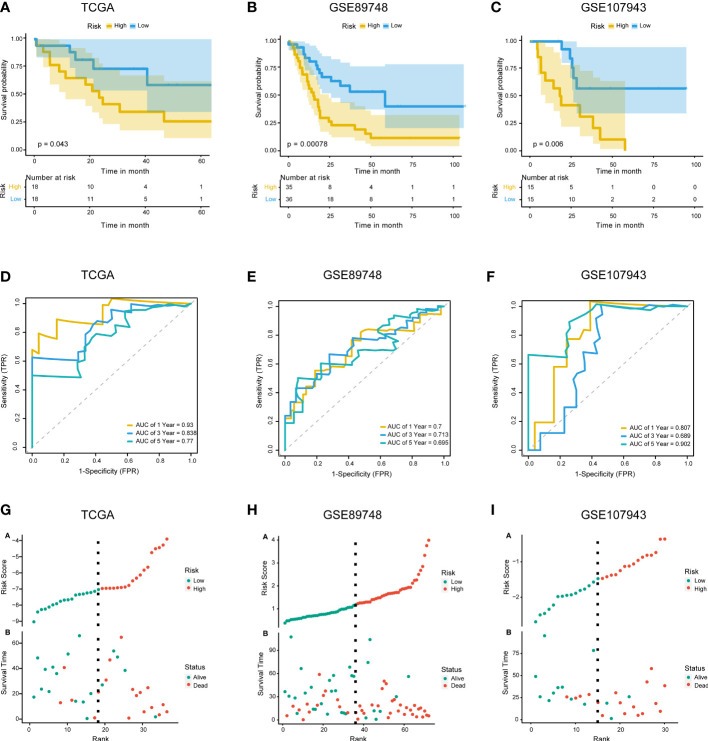
Construction and validation of the prognostic model. **(A–C)**. KM survival curves for overall survival in TCGA training cohort **(A)**, GSE89748 validation cohort **(B)**, and GSE107943 validation cohort **(C)**. **(D–F)** Time-dependent ROC curve of TCGA cohort **(D)**, GSE89748 cohort **(E)**, and GSE107943 cohort **(F)**. Sensitivity (TRP) = TP/(TP+FN) and false positive prediction rate (FPR) (1-specificity = FP/(FP+TN)) were used as the y-axis and x-axis variables, where TPs (true positives) are positive predictions which belong to gold standard positives (GSPs), FNs (false negatives) are negative predictions which belong to GSPs.TP, true positive; FP, false positive; TN, true negative. **(G–I)** Distributions of risk scores and OS status are shown for TCGA cohort **(G)**, GSE89748 cohort **(H)**, and GSE107943 cohort **(I)**.

The 67 DENRGs were subjected to univariate Cox proportional hazard regression analysis. Five prognostic genes (*PYGB*, *IFNGR2*, *TICAM1*, *STAT6*, and *VPS4B*) were selected and further analyzed using multivariate Cox proportional hazards regression analysis. The coefficients from the multivariate Cox proportional hazards regression model were used to evaluate the potential prognostic factors. Risk scores were also calculated in TCGA (training cohort), GSE89748 (validation cohort 1), and GSE107943 (validation cohort 2) according to the prognostic gene expression values and their regression coefficients. Taking the median risk score of the samples as the cutoff value, CHOL patients were divided into high- and low-risk groups. Survival analysis showed that the low-risk group exhibited a better outcome in TCGA (log-rank test p-value < 0.05) (**Figure 3A**), GSE89748 (log-rank test p-value < 0.001) (**Figure 3B**), and GSE107943 (log-rank test p-value < 0.001) (**Figure 3C**). Next, we performed 1-, 3-, and five-year time-dependent ROC analyses in three independent datasets (TCGA, GSE89748, and GSE107943). The results show that the AUC of time-dependent ROC curves was greater than 0.6 in all datasets (**Figures 3D–F**). Notably, the AUC of the 1-year time-dependent ROC exceeded 0.7, indicating that the prognostic risk score had good prediction abilities. A risk plot also illustrated the distributions of the risk scores and the OS status in the three dependent datasets (**Figures 3G–I**). It is worth mentioning that the increase in the prognostic risk score and the number of death events in patients increased.

### 3.4 Construction and evaluation of the nomogram model

**Figure 4:**
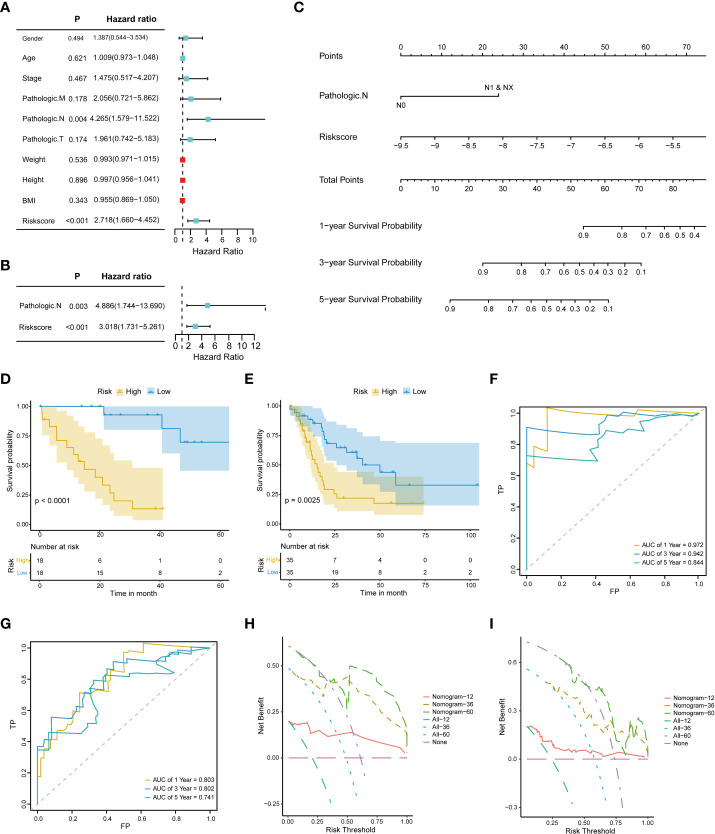
Construction and evaluation of the nomogram model. **(A)** Univariate Cox proportional hazard regression analysis of the clinical characteristics. **(B)** Multivariate Cox proportional hazard regression analysis of selected clinical characteristics. **(C)** Prediction of 1-, 3-, and 5-year survival probabilities for CHOL patients using the nomogram model. **(D, E)**. Survival curve for the low-risk and high-risk subgroups in the training dataset and the validation dataset. **(F, G)**. Time-dependent ROC curves of the training cohort and the validation cohort. Sensitivity (TRP) = TP/(TP+FN) and false positive prediction rate (FPR) (1-specificity = FP/(FP+TN)) were used as the y-axis and x-axis variables, where TPs (true positives) are positive predictions which belong to gold standard positives (GSPs), FNs (false negatives) are negative predictions which belong to GSPs.TP, true positive; FP, false positive; TN, true negative **(H, I)**. DCA curves of the training cohort and the validation cohort.

Univariate and multivariate Cox regression analyses were performed on the clinical characteristics and risk scores in TCGA to explore the prognostic factors of patients. The results show that two factors, the risk score and pathologic N, were significantly associated with patient prognosis (p < 0.05) (**Figures 4A, B**). Subsequently, a nomogram model for predicting 1-, 3-, and 5-year OS was constructed, which integrated the two factors that were significantly correlated with prognosis: pathologic N and the prognostic risk score (**Figure 4C**). Besides, we established calibration curves to verify the effectiveness of nomogram model for predicting the rates of OS for CHOL patients at 1, 3, and 5 years. The results showed that the calibration curves displayed a suitable agreement between the prediction by nomogram and actual survival (**Figure S2**).

A risk classification system was then constructed based on the risk scores calculated from the nomogram model for each CHOL patient. Using this system, the enrolled patients were divided into low- and high-risk groups. The outcomes show that the low-risk group had the best prognosis, and the high-risk group had the worst prognosis (**Figures 4D, E**). Time-dependent ROC analysis showed that the 1-, 3-, and 5-year nomogram models exhibited AUC > 0.7, and even the 1- and 3-year time-dependent ROC exhibited AUC > 0.8 (**Figures 4F, G**). We further used decision curve analysis (DCA) to evaluate the clinical predictive models. The results showed that the DCA curves at 1, 3, and 5 years remained above the gray and black lines between 0 and 1.0, in TCGA CHOL and GSE89748 datasets, suggesting that CHOL patients may benefit from decisions based on the prognostic model (**Figures 4H, I**).

### 3.5 Identification of DEGs and functional enrichment analysis

**Figure 5:**
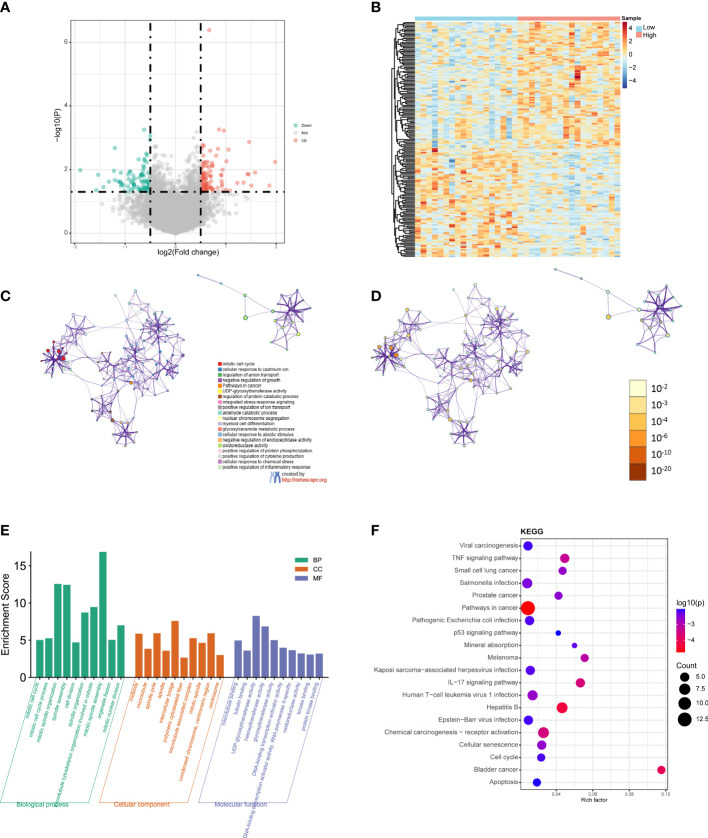
GO and KEGG functional enrichment analyses between the low- and high-risk groups. **(A)** Volcano plot of the DEGs. Red represents upregulated genes (Up), blue represents downregulated genes (Down), and gray represents not significantly different genes (Not). **(B)** Heatmap of the DEGs between the high-risk group and the low-risk group. Red indicates the high-risk group (High) and blue indicates the low-risk group (Low). **(C)** A network diagram of the top 20 enriched biological functions. Cluster IDs are represented using different colors, while enriched terms are indicated by nodes. **(D)** Twenty enriched biological functions are shown in this network diagram, and the p-values are displayed as different colors, while the enriched terms are indicated as nodes. **(E)** Bar-plot of GO terms, with the height of the column indicating the enrichment score. **(F)** Dot plot of the KEGG enrichment analyses results. The dot scale represents the number of genes in each KEGG term; the depth of the dot color represents the p-value.

Next, we performed differential expression analysis on TCGA CHOL datasets of the high- and low-risk groups to obtain DEGs. According to the screening thresholds (|log2FC| > 0.5 and p < 0.05), 179 DEGs were identified in the high- and low-risk groups, including 96 upregulated genes and 83 downregulated genes (**Figure 5A**). In addition, the heatmaps revealed that the expression patterns of genes were also classified into two categories, along with the high- and low-risk groups (**Figure 5B**).

GO and KEGG functional enrichment analyses of the DEGs were performed using Metascape. The top 20 enriched biological function terms were displayed in the network diagrams according to their enrichment scores (**Figures 5C, D**). The GO analysis results show that the DEGs were mainly associated with mitotic cell cycle, mitotic spindle organization, mitotic spindle assembly, intercellular bridge, polymeric cytoskeletal fiber, hexosyltransferase activity, DNA, Binding transcription activator activity, and protein kinase binding (**Figure 5E**). According to the KEGG analysis results, pathways in cancer, viral carcinogenesis, TNF signaling pathway, *Salmonella* infection, pathogenic *Escherichia coli* infection, IL−17 signaling pathway, hepatitis B, chemical carcinogenesis-receptor activation, and apoptosis were significantly enriched (**Figure 5F**). The detailed results are summarized in **Table S2**.

**Figure 6:**
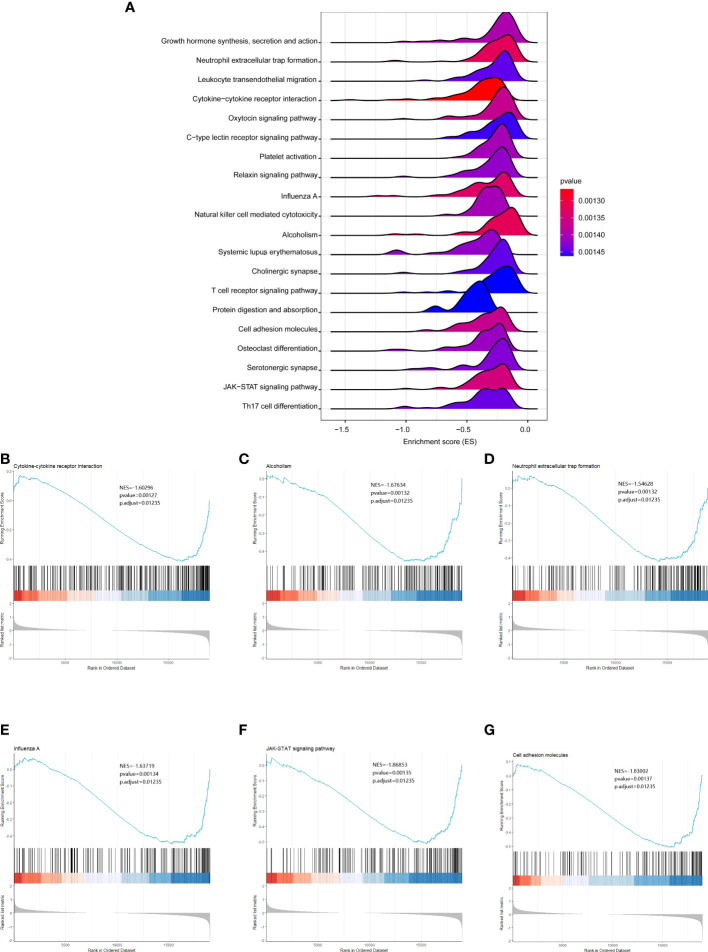
GSEA analysis results between the low- and high-risk groups. **(A)** Ridgeline plots showing the top 20 enriched KEGG terms in the low- and high-risk groups. ES (enrichment score) reflected the correlation between the gene set and the sample. B-G. Cytokine-cytokine receptor interaction **(B)**, alcoholism **(C)**, neutrophilic extracellular trap formation **(D)**, influenza A **(E)**, JAK-STAT signaling pathway **(F)**, and cell adhesion molecules **(G)** were significantly enriched in the low-risk group.

To further analyze the functional implications of the five necroptosis gene signatures in CHOL, we performed GSEA of TCGA CHOL expression profiles according to low- and high-risk groups. As shown in **Figure 6A**, the ridgeline plot reveals the top 20 enriched KEGG terms in the low- and high-risk groups. These results show that cytokine-cytokine receptor interaction, alcoholism, neutrophilic extracellular trap formation, influenza A, JAK-STAT signaling pathway, and cell adhesion molecules were significantly enriched in the low-risk group (**Figures 6B–G**). Detailed GSEA results are presented in **Table S3**.

### 3.6 Multi-omics analysis based on prognostic risk scores

**Figure 7:**
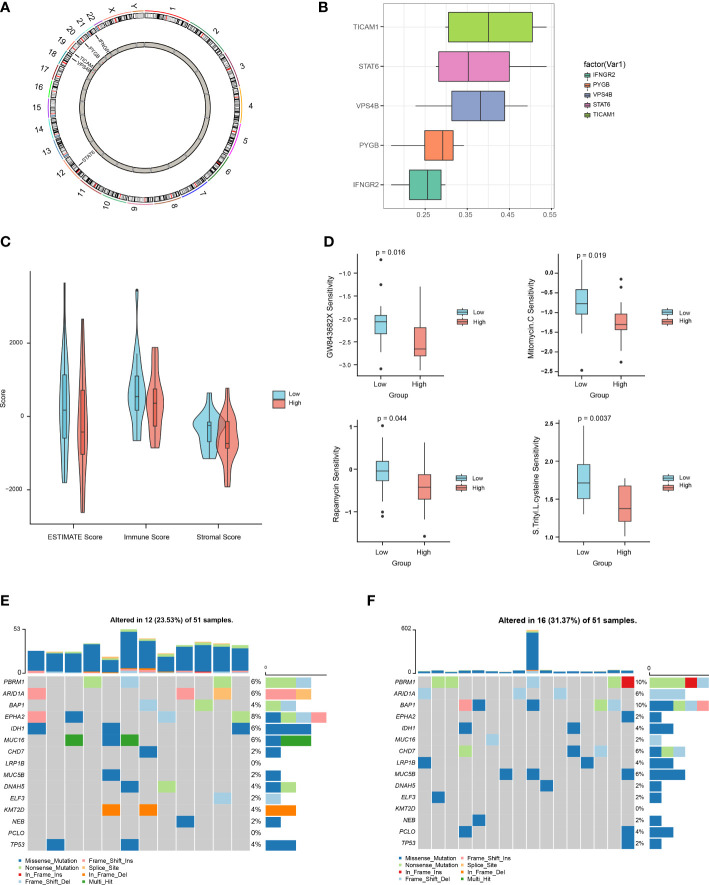
Multi-omics analysis based on the prognostic risk scores. **(A)** Chromosome localization map of necroptosis prognosis genes. **(B)** Friends analysis of necroptosis prognosis genes. **(C)** Differences in ESTIMATE, immune, and stromal scores between the high- and low-risk groups. **(D)** Differences between the high- and low-risk groups in terms of drug sensitivity. **(E, F)**. Oncoplot mutations in the low- and high-risk groups.

We then used the R package “Rcircos” to map the chromosomal locations of the above five NRGs. The gene chromosome location diagram revealed that *PYGB, IFNGR2, TICAM1, STAT6*, and *VPS4B* are located on chr20, chr21, chr19, chr12, and chr18, respectively (**Figure 7A**). Friends analyses of the necroptosis-associated prognostic genes revealed that *TICAM1* was the most important term (**Figure 7B**). In the low-risk group, the ESTIMATE, immune, and stromal scores were all higher than those in the high-risk group, according to violin plots (**Figure 7C**). The therapeutic effects of the four drugs on CHOL are shown as boxplots. The results show that the sensitivity to GW843682X, mitomycin C (MMC), rapamycin, and S-trityl-L-cysteine (STLC) was significantly higher in the low-risk group than in the high-risk group (**Figure 7D**). The oncoplot demonstrated different mutation patterns between the high- and low-risk groups (**Figures 7E, F**).

**Figure 8:**
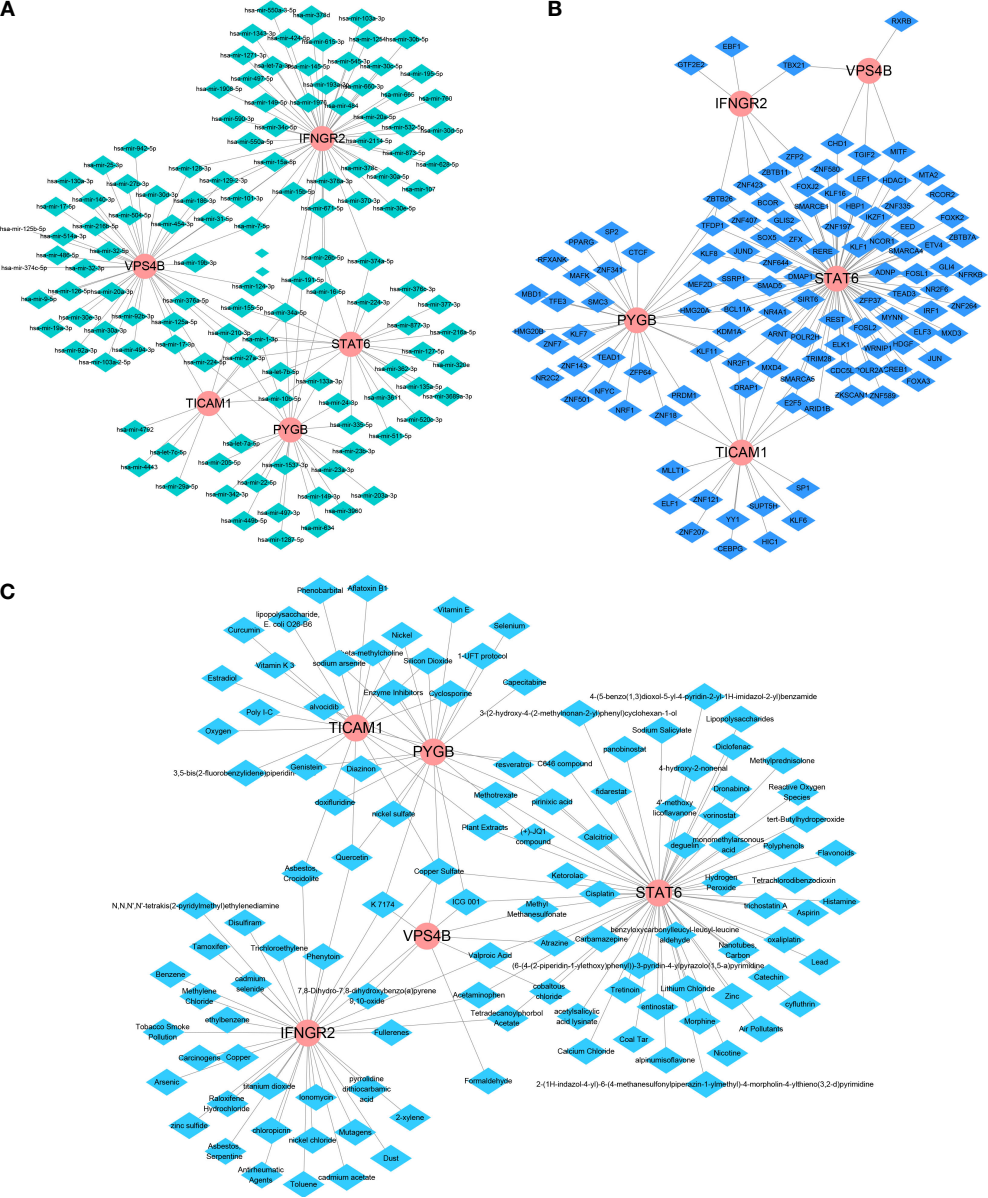
An integrated network of TFs, miRNAs, and chemicals target the necroptosis prognosis genes. **(A)** The integrated network diagrams between the five NRGs and miRNAs. **(B)** The integrated network diagrams between the five NRGs and TFs. **(C)** The integrated network diagrams between the five NRGs and potential chemical modulators.

We further used Network-Analyst to obtain network diagrams of the interaction between the five NRGs and miRNAs, transcription factors (TFs), and potential chemicals. The results show that 124 miRNAs targeting the five necroptosis prognosis genes fit a network diagram (**Figure 8A**). In the TF-necroptosis prognosis gene network diagram, 114 TFs were observed (**Figure 8B**). A total of 117 potential chemical targets were identified using Network Analyst (**Figure 8C**).

### 3.7 Analysis of immune cell infiltration and its correlation with the five NRGs

**Figure 9:**
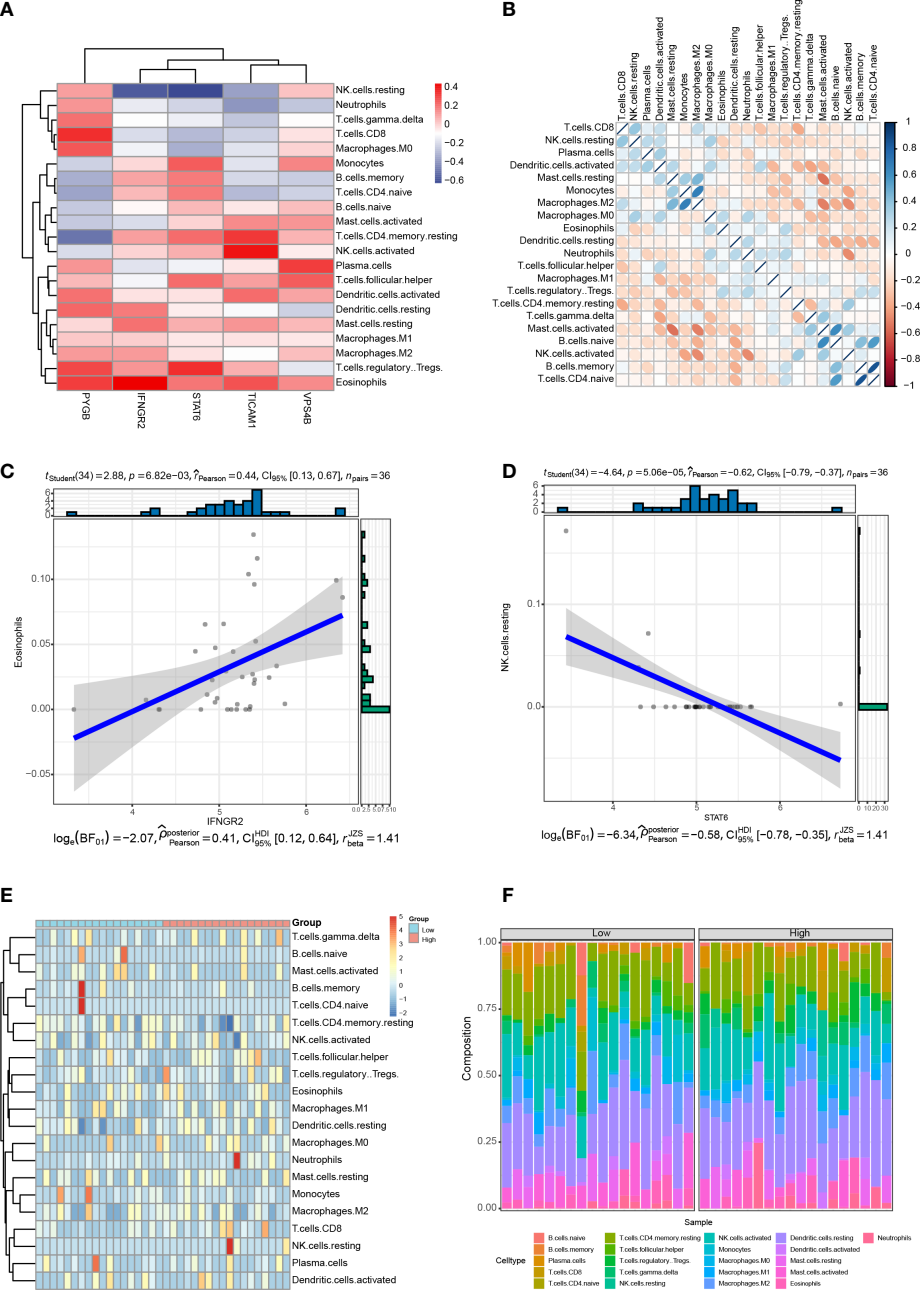
Correlation between the five NRGs and immune cell infiltration of CHOL. **(A)** Correlation analyses between 22 different immune cell types and the five NRGs in the CHOL group. Red color represents positive correlation whereas blue color indicates negative correlation. **(B)** Heatmap of the correlation between 22 different immune cell types. Positive correlations are in red and negative correlations are in blue. The darker the color, the stronger the correlation. **(C)** Correlation analysis between *IFNGR2* and Eosinophils. **(D)** Correlation analysis between *STAT6* and resting NK cells. **(E)** A heatmap showing the difference in immune cell infiltration between the high-risk and low-risk groups. **(F)** Box plot of the proportion of immune cell infiltration between the high-risk and low-risk groups.

Immune cell infiltration is a critical factor in the progression of CHOL, and it significantly affects the survival rate of patients with CHOL (9, 33). We analyzed the relationship between the expression of the five NRGs and infiltration of 22 immune cell types in CHOL. The results show that *IFNGR2* and *STAT6* were negatively correlated with resting natural killer (NK) cells, whereas *PYGB* was significantly positively correlated with CD8^+^ T cells, M0 macrophages, Tregs, and eosinophils. *TICAM1* was positively correlated with resting central memory CD4^+^ T cells and activated NK cells, and *VPS4B* was positively correlated with plasma cells and T follicular helper cells. *STAT6* expression positively correlated with monocytes and Tregs (**Figure 9A**). A heatmap of the correlation between the 22 different immune cell types indicates that M2 macrophages had a clear positive correlation with monocytes; naive B cells had a clear positive correlation with activated mast cells and naive CD4^+^ T cells; memory B cells had a clear positive correlation with naive CD4^+^ T cells, while activated mast cells exhibited obvious inverse correlations with resting mast cells and M2 macrophages; activated NK cells had an obvious inverse correlation with monocytes, M2 macrophages, and neutrophils (**Figure 9B**). The strongest positive correlation was observed between *IFNGR2* and eosinophils (**Figure 9C**). In contrast, *STAT6* exhibited the strongest negative correlation with resting NK cells (**Figure 9D**). The high-risk and low-risk groups exhibited significantly different levels of immune cell infiltration in the heatmap (**Figure 9E**). The boxplot indicates that there was a significant difference in the proportion of immune cells between the high- and low-risk groups. B cells accounted for a higher proportion in the low-risk group, whereas T cells accounted for a higher proportion in the high-risk group (**Figure 9F**).

### 3.8 Validation of the five NRGs expressions in CHOL tissue samples

**Figure 10:**
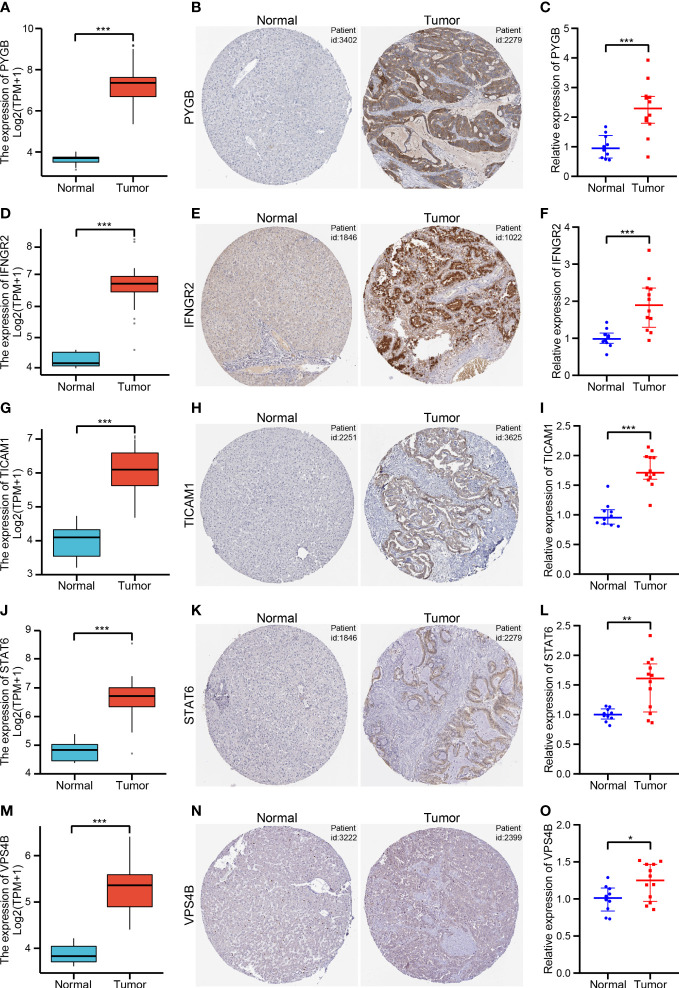
Validation of the five NRGs expressions in CHOL tissue samples **(A, D, G, J, M)**. The expression levels of *PYGB* **(A)**, *IFNGR2* **(D)**, *TICAM1* **(G)**, *STAT6* **(J)** and *VPS4B* **(M)** between CHOL and normal samples using the TCGA database. **(B, E, H, K, N)**. Immunohistochemistry of *PYGB* **(B)**, *IFNGR2* **(E)**, *TICAM1* **(H)**, *STAT6* **(K)** and *VPS4B* **(N)** in CHOL and normal samples from the HPA database. **(C, F, I, L, O)**. Relative expression of *PYGB* **(C)**, *IFNGR2* **(F)**, *TICAM1* **(I)**, *STAT6* **(L)** and *VPS4B* **(O)** was detected by qRT-PCR in CHOL and normal samples. *p < 0.05, **p < 0.01, ***p < 0.001.

We further validated the expression of five NRGs using the TCGA database, HPA database and our clinical data. TCGA database results showed that *PYGB* (**Figure 10A**), *IFNGR2* (**Figure 10D**), *TICAM1* (**Figure 10G**), *STAT6* (**Figure 10J**) and *VPS4B* (**Figure 10M**) were expressed at high levels in CHOL tissues. Based on the protein expression data from the HPA, the immunohistochemistry results confirmed that the protein expression levels of *PYGB* (**Figure 10B**), *IFNGR2* (**Figure 10E**), *TICAM1* (**Figure 10H**), *STAT6* (**Figure 10K**) and *VPS4B* (**Figure 10N**) were higher in CHOL tissues than normal hepatobiliary duct tissues. Finally, we detected their expression levels in 10 non-tumor hepatobiliary duct tissues and 12 CHOL tissues by using RT-qPCR assay. The results showed that the expression levels of *PYGB* (**Figure 10C**), *IFNGR2* (**Figure 10F**), *TICAM1* (**Figure 10I**), *STAT6* (**Figure 10L**) and *VPS4B* (**Figure 10O**) in CHOL tissues showed an overall upward trend compared with non-tumor hepatobiliary duct tissues.

## 4 Discussion

CHOL is the second most common primary malignancy of the liver after hepatocellular carcinoma, with a steady increase in its incidence and mortality rate (1). When hepatocytes die due to necroptosis, the necroptosis-dominated microenvironment leads to the development of CHOL. Recent studies have also found that necroptosis plays a pivotal role in regulating carcinogenesis, cancer subtypes, immunity, metastasis, and anticancer treatments (2, 3). The molecular mechanism by which necroptosis is involved in the genesis and development of CHOL remains unclear, however.

In this study, we focused on developing and validating a prognostic signature for CHOL using necroptosis-related genes. First, 65 DENRGs were identified between the CHOL and control groups. Secondly, five genes (*PYGB*, *IFNGR2*, *TICAM1*, *STAT6*, and *VPS4B*) were identified as prognostic signatures based on multivariate Cox regression analysis. The Kaplan–Meier survival curves in TCGA also indicate that the low-risk group had significantly longer patient survival than the high-risk group. The survival results were also validated independently using the GSE89748 and GSE107943 datasets. In addition, the nomogram model was highly discriminatory for OS based on the pathologic N and risk score. Moreover, patients with high-risk scores experienced higher immune cell infiltration, drug resistance, and more somatic mutations. In summary, these results suggest that the five genes related to necroptosis play prominent roles in modulating drug resistance, somatic mutations, and the tumor microenvironment, indicating that these risk signatures were highly robust and accurate in predicting the prognosis of patients with CHOL.

Our prognostic signature consists of five genes, *PYGB*, *IFNGR2*, *TICAM1*, *STAT6*, and *VPS4B*, each of which plays a critical role in necroptosis and tumor progression. *PYGB* codes for the protein glycogen phosphorylase B, which is found predominantly in the brain (34). *PYGB* has been reported to be involved in the progression of gastric and liver cancers (35, 36). *IFNGR2* codes for the IFN-γ receptor, which has been found to mediate a non-immunogenic tumor phenotype associated with checkpoint inhibitor resistance in renal carcinoma (37, 38). *TICAM1* codes for an essential necrosome adaptor protein that functions as an essential signal transducer in Toll-like receptor (TLR) 3 and TLR4 signaling pathways (39). It has been reported that *TLR3/TICAM1* signaling is involved in tumor cell RIP3-dependent necroptosis, which contributes to immune effector-mediated tumor elimination (38). In our study, *TICAM1* was highly expressed in the CHOL group and was positively correlated with resting central memory CD4^+^ T cells and NK cell activation, suggesting that the *TICAM1* gene product is involved in the tumor microenvironment. STAT6 is highly expressed in a variety of human cancers and has been suggested to induce apoptosis and growth inhibition of hepatocellular carcinoma-derived cells by lowering RANKL expression (40). *VPS4B* codes for a protein that is involved in autophagy that can reduce the sensitivity of T cell-mediated tumor cell lysis by lowering granzyme B content, and it is an essential factor required for escaping CD8^+^ T cell-mediated killing in tumors (41, 42). In keeping with this, *VPS4B* was negatively correlated with follicular helper T cells and was found to be highly expressed in CHOL in our study. Overall, our study investigated the prognostic value of five necroptosis-related markers in CHOL. Further in-depth experimental research is needed to explore the potential regulatory effects of this gene set on necroptosis.

In recent years, regulation of the tumor immune microenvironment through immunotherapy has revolutionized cancer treatment (43, 44). Numerous studies have confirmed that immunotherapy based on alteration of the tumor immune microenvironment can affect tumor metastasis, immune escape, and immunotherapy resistance by modifying the immune response (45–47). For instance, a study has suggested that increasing the number or function of NK cells may be a promising approach for the treatment of CHOL (48). Our study found a negative correlation of *STAT6* with resting NK cells, thus suggesting that STAT6 is a potential immunotherapy target. Higher infiltration of M1 and M2 macrophages is related to a poor prognosis by accelerating tumor progression through the secretion of pro-angiogenic factors, activation of the Wnt/β-catenin pathway, and suppression of the antitumor functions of T cells (49). In our study, the high-risk group, which had a poor prognosis, had a higher level of M0 macrophage infiltration, indicating that a greater number of non-activated macrophages were present.

The DEGs between the high- and low-risk groups were enriched in immune-related biological processes and pathways. The five genes involved in our prognostic signature correlated with different levels of immune cell infiltration, such as NK cells, T cells, monocytes, M0 macrophages, and plasma cells. Our results show that, based on the gene signature, there were clear differences in the degree of immune cell infiltration between the high-risk and low-risk groups. The high-risk group tended to exhibit a higher proportion of multiple types of T cells, whereas the low-risk group exhibited a higher proportion of multiple types of B cells. In addition, the low-risk group had higher stromal, immune, and ESTIMATE scores than the high-risk group. In summary, our prognostic signature for CHOL based on necroptosis-related genes could reflect the tumor immune microenvironment of CHOL, which could potentially contribute to personalized immunotherapy and targeted therapy for patients with CHOL.

According to previous studies examining genomic alterations, gene mutations in CHOL usually result in poor outcomes (50). Our study also demonstrated that necroptosis-related genes were positively correlated with genomic alterations, and the high-risk group (mutation rate: 31.37%) exhibited more somatic mutations than the low-risk group (mutation rate: 23.53%). In particular, missense mutations were by far the most predominant mutation type found in CHOL. Moreover, *PBRM1* and *BAP1* exhibited significantly increased mutation rates and multiple mutation types in the high-risk group. In addition, the high-risk group exhibited higher levels of resistance to treatment with GW843682X, mitomycin C, rapamycin, and S-trityl-L-cysteine. These results show that our prognostic signature could be used as a potential predictor of the efficacy of medical treatment for CHOL. Moreover, the occurrence of drug resistance may be reduced by regulation of this signature, which could potentially lead to new breakthroughs in the choice of individual therapeutic strategies.

However, the current study has some limitations. First, the data gathered were from public databases, which were limited in sample size. Future research with a larger sample size is needed to overcome these limitations. Secondly, the identified genes have complex functions and molecular mechanisms that need to be further verified in cellular and animal models. Finally, more detailed clinical follow-up data are required to confirm the value of our prognostic model.

## 5 Conclusion

In this study, we shed further light on the role of necroptosis in the prognosis of CHOL. Our results indicate that the prognostic model derived from the five NRGs can accurately predict the prognosis of patients with CHOL. Furthermore, the risk score derived from the necroptosis model is associated with important biological functions and is clinically significant. Therefore, the predictive signature of the five NRGs may help devise individualized treatments for patients in the future.

## Data availability statement

The datasets presented in this study can be found in online repositories. The names of the repository/repositories and accession number(s) can be found within the article/**Supplementary Material**.

## Ethics statement

The studies involving human participants were reviewed and approved by The Ethics Committee of the First Affiliated Hospital of Zhengzhou University (Approval Number: SS-2019-018). The patients/participants provided their written informed consent to participate in this study. Written informed consent was obtained from the individual(s) for the publication of any potentially identifiable images or data included in this article.

## Author contributions

LX and ZG designed the project and analyzed the data. XG downloaded the data and drafted the manuscript. JX collected and analyzed the data. All authors contributed to the article and approved the submitted version.
