# Supplementary material for: Identification of a necroptosis-related gene signature as a novel prognostic biomarker of cholangiocarcinoma
Source: Front Immunol. 2023 Mar 2;14:1118816. doi: 10.3389/fimmu.2023.1118816 (PMC10017743; doi:10.3389/fimmu.2023.1118816)
Supplement: Supplementary file 1 [file DataSheet_1.docx]

# Supplementary figure legends

**Figure S1. A and B. The expression of 65 DENRGs in normal and CHOL**.

**Figure S2. Calibration plot of the nomogram for predicting the probability of OS at 1, 3, and 5 years.** The abscissa is the probability of the prognosis predicted by the model (0–1 indicates that the probability of the event occurring is 0–100%) and the ordinate is the actual observed prognosis. The gray diagonal is the ideal case.
