# Supplementary figures and images for: Identification of a necroptosis-related gene signature as a novel prognostic biomarker of cholangiocarcinoma
Source: Front Immunol. 2023 Mar 2;14:1118816. doi: 10.3389/fimmu.2023.1118816 (PMC10017743; doi:10.3389/fimmu.2023.1118816)

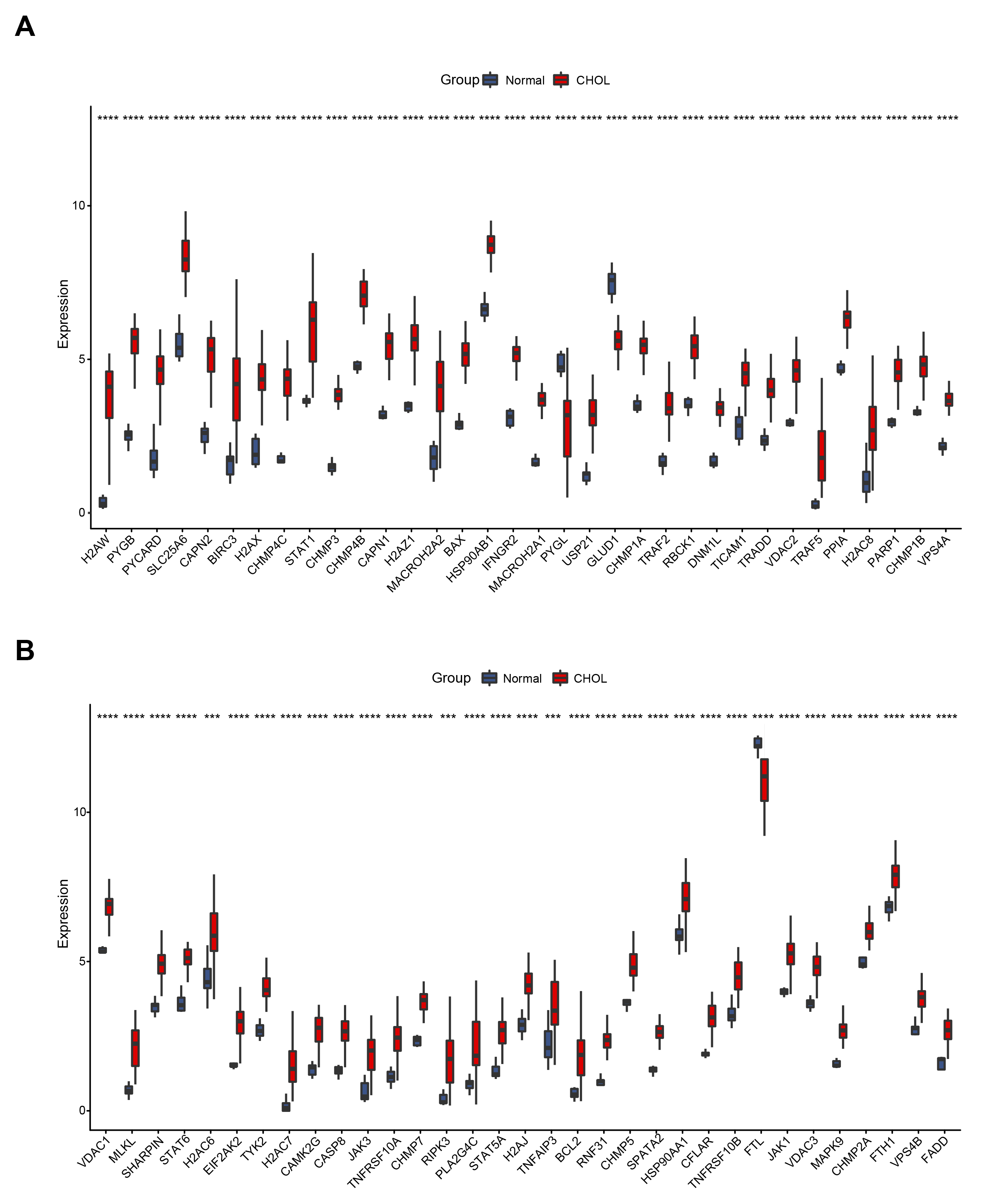

Supplement: Supplementary file 2 [file Image_1.tif]

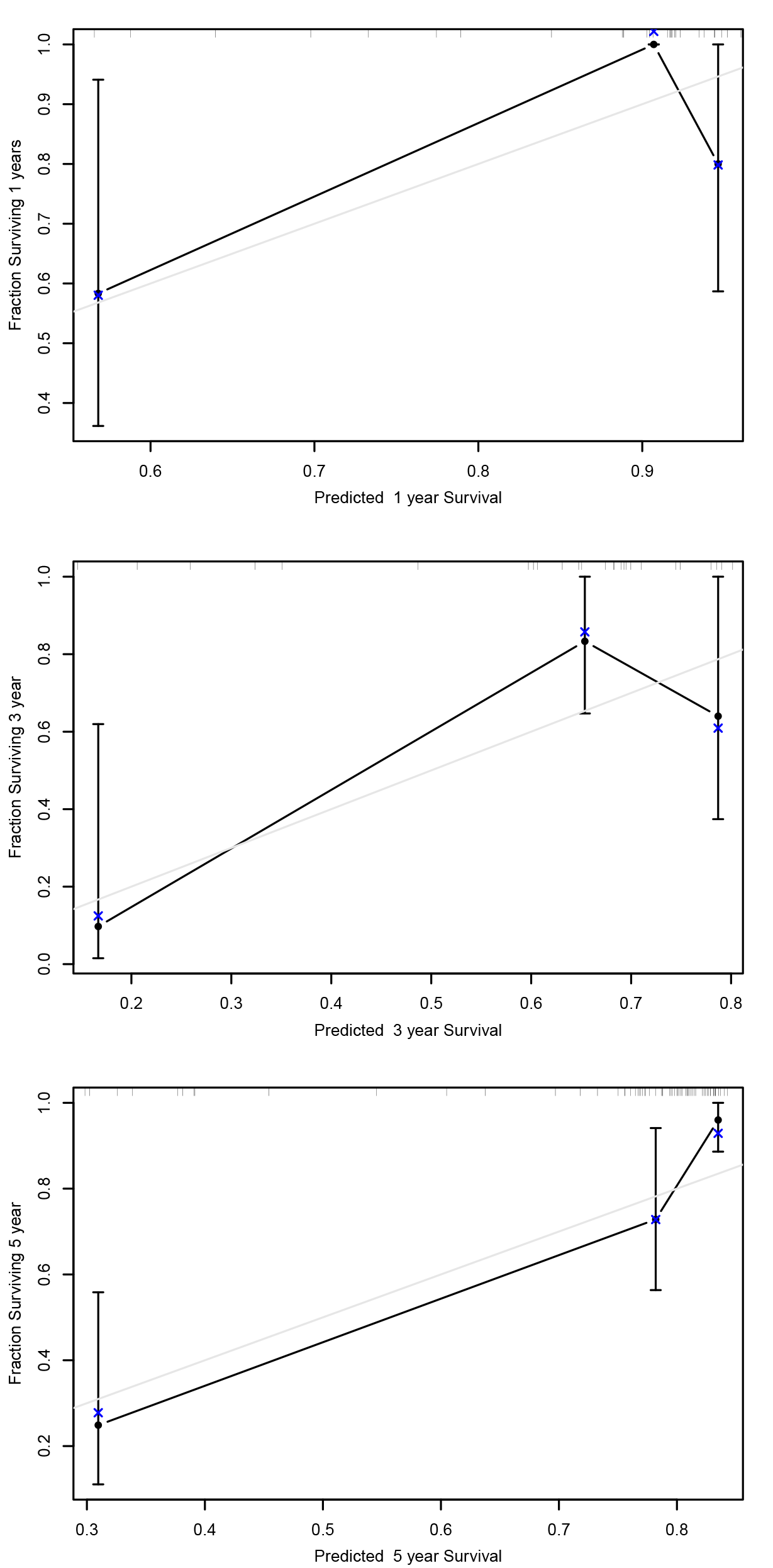

Supplement: Supplementary file 3 [file Image_2.tif]
