# Supplementary material for: Identification of a necroptosis-related gene signature as a novel prognostic biomarker of cholangiocarcinoma
Source: Front Immunol. 2023 Mar 2;14:1118816. doi: 10.3389/fimmu.2023.1118816 (PMC10017743; doi:10.3389/fimmu.2023.1118816)
Supplement: Supplementary file 4 [file Table_1.docx]

**Supplementary Table S1: Top enrichment function obtained by GO and KEGG analysis based on differentially expressed necroptosis-related genes.**

| Category | GO | Description | Count | P.adjust |
| --- | --- | --- | --- | --- |
| GO Biological Processes | GO:0097300 | programmed necrotic cell death | 13 | 2.36E-18 |
| GO Biological Processes | GO:0061952 | midbody abscission | 10 | 3.49E-18 |
| GO Biological Processes | GO:0070265 | necrotic cell death | 13 | 2.40E-17 |
| GO Biological Processes | GO:1902410 | mitotic cytokinetic process | 10 | 1.73E-16 |
| GO Biological Processes | GO:0046755 | viral budding | 10 | 2.08E-16 |
| GO Biological Processes | GO:0019068 | virion assembly | 11 | 2.08E-16 |
| GO Biological Processes | GO:0070266 | necroptotic process | 11 | 1.12E-15 |
| GO Biological Processes | GO:0036258 | multivesicular body assembly | 10 | 1.30E-15 |
| GO Biological Processes | GO:0036257 | multivesicular body organization | 10 | 1.70E-15 |
| GO Biological Processes | GO:0039702 | viral budding via host ESCRT complex | 9 | 3.35E-15 |
| GO Cellular Components | GO:0000815 | ESCRT III complex | 7 | 6.63E-14 |
| GO Cellular Components | GO:0036452 | ESCRT complex | 7 | 1.74E-10 |
| GO Cellular Components | GO:0000786 | nucleosome | 9 | 4.67E-09 |
| GO Cellular Components | GO:0044815 | DNA packaging complex | 9 | 6.73E-09 |
| GO Cellular Components | GO:0032993 | protein-DNA complex | 10 | 4.47E-08 |
| GO Cellular Components | GO:0046930 | pore complex | 5 | 3.06E-07 |
| GO Cellular Components | GO:0000790 | nuclear chromatin | 11 | 1.12E-06 |
| GO Cellular Components | GO:0031902 | late endosome membrane | 7 | 6.51E-06 |
| GO Cellular Components | GO:0010008 | endosome membrane | 11 | 9.55E-06 |
| GO Cellular Components | GO:0045121 | membrane raft | 9 | 1.57E-05 |
| GO Molecular Functions | GO:0032813 | tumor necrosis factor receptor superfamily binding | 7 | 8.58E-08 |
| GO Molecular Functions | GO:0005164 | tumor necrosis factor receptor binding | 6 | 1.78E-07 |
| GO Molecular Functions | GO:0005126 | cytokine receptor binding | 10 | 8.50E-06 |
| GO Molecular Functions | GO:0044389 | ubiquitin-like protein ligase binding | 10 | 1.27E-05 |
| GO Molecular Functions | GO:0002020 | protease binding | 7 | 2.38E-05 |
| GO Molecular Functions | GO:0031625 | ubiquitin protein ligase binding | 9 | 5.20E-05 |
| GO Molecular Functions | GO:0008234 | cysteine-type peptidase activity | 7 | 0.00015132 |
| GO Molecular Functions | GO:0051400 | BH domain binding | 3 | 0.00015132 |
| GO Molecular Functions | GO:0070513 | death domain binding | 3 | 0.00015132 |
| GO Molecular Functions | GO:0019903 | protein phosphatase binding | 6 | 0.000329546 |
| KEGG Pathway | hsa04217 | Necroptosis | 67 | 4.40E-120 |
| KEGG Pathway | hsa04621 | NOD-like receptor signaling pathway | 23 | 6.33E-20 |
| KEGG Pathway | hsa04210 | Apoptosis | 14 | 1.70E-10 |
| KEGG Pathway | hsa05164 | Influenza A | 15 | 2.04E-10 |
| KEGG Pathway | hsa05162 | Measles | 13 | 2.22E-09 |
| KEGG Pathway | hsa04668 | TNF signaling pathway | 12 | 2.22E-09 |
| KEGG Pathway | hsa05161 | Hepatitis B | 13 | 1.12E-08 |
| KEGG Pathway | hsa05169 | Epstein-Barr virus infection | 14 | 1.36E-08 |
| KEGG Pathway | hsa05132 | Salmonella infection | 15 | 1.93E-08 |
| KEGG Pathway | hsa04613 | Neutrophil extracellular trap formation | 13 | 5.64E-08 |
