# Supplementary material for: Identification of a necroptosis-related gene signature as a novel prognostic biomarker of cholangiocarcinoma
Source: Front Immunol. 2023 Mar 2;14:1118816. doi: 10.3389/fimmu.2023.1118816 (PMC10017743; doi:10.3389/fimmu.2023.1118816)
Supplement: Supplementary file 5 [file Table_2.docx]

| Category | GO | Description | Count | LogP |
| --- | --- | --- | --- | --- |
| GO Biological Processes | GO:0000278 | mitotic cell cycle | 18 | -7.613288077 |
| GO Biological Processes | GO:1903047 | mitotic cell cycle process | 16 | -7.085171355 |
| GO Biological Processes | GO:0007052 | mitotic spindle organization | 7 | -5.819421423 |
| GO Biological Processes | GO:0051225 | spindle assembly | 7 | -5.788340684 |
| GO Biological Processes | GO:0051301 | cell division | 14 | -5.690475232 |
| GO Biological Processes | GO:0007051 | spindle organization | 8 | -5.369498097 |
| GO Biological Processes | GO:1902850 | microtubule cytoskeleton organization involved in mitosis | 7 | -4.994773592 |
| GO Biological Processes | GO:0090307 | mitotic spindle assembly | 5 | -4.927717865 |
| GO Biological Processes | GO:0048285 | organelle fission | 10 | -4.468228556 |
| GO Biological Processes | GO:0140014 | mitotic nuclear division | 7 | -4.153096258 |
| GO Cellular Components | GO:0030496 | midbody | 7 | -3.685870617 |
| GO Cellular Components | GO:0005874 | microtubule | 10 | -3.508696442 |
| GO Cellular Components | GO:0000922 | spindle pole | 6 | -3.24766694 |
| GO Cellular Components | GO:0005819 | spindle | 9 | -2.986208559 |
| GO Cellular Components | GO:0045171 | intercellular bridge | 4 | -2.705702441 |
| GO Cellular Components | GO:0099513 | polymeric cytoskeletal fiber | 12 | -2.681742966 |
| GO Cellular Components | GO:0005875 | microtubule associated complex | 5 | -2.567050946 |
| GO Cellular Components | GO:0072686 | mitotic spindle | 5 | -2.329013184 |
| GO Cellular Components | GO:0000779 | condensed chromosome, centromeric region | 6 | -3.24766694 |
| GO Cellular Components | GO:0005813 | centrosome | 11 | -2.90511599 |
| GO Molecular Functions | GO:0008017 | microtubule binding | 8 | -3.635332024 |
| GO Molecular Functions | GO:0015631 | tubulin binding | 8 | -2.722619717 |
| GO Molecular Functions | GO:0008194 | UDP-glycosyltransferase activity | 7 | -4.615090003 |
| GO Molecular Functions | GO:0016758 | hexosyltransferase activity | 8 | -4.61066694 |
| GO Molecular Functions | GO:0016757 | glycosyltransferase activity | 8 | -3.656974776 |
| GO Molecular Functions | GO:0001228 | DNA-binding transcription activator activity, RNA polymerase II-specific | 10 | -3.335159499 |
| GO Molecular Functions | GO:0001216 | DNA-binding transcription activator activity | 11 | -3.942623663 |
| GO Molecular Functions | GO:0016491 | oxidoreductase activity | 14 | -3.869287876 |
| GO Molecular Functions | GO:0019900 | kinase binding | 14 | -3.662164473 |
| GO Molecular Functions | GO:0019901 | protein kinase binding | 13 | -3.595127919 |
| KEGG Pathway | hsa04978 | Mineral absorption | 3 | -2.261991599 |
| KEGG Pathway | hsa05200 | Pathways in cancer | 13 | -4.729720983 |
| KEGG Pathway | hsa05219 | Bladder cancer | 4 | -3.992038451 |
| KEGG Pathway | hsa05218 | Melanoma | 4 | -3.047199839 |
| KEGG Pathway | hsa05222 | Small cell lung cancer | 4 | -2.653094307 |
| KEGG Pathway | hsa04218 | Cellular senescence | 5 | -2.614529974 |
| KEGG Pathway | hsa05215 | Prostate cancer | 4 | -2.569592776 |
| KEGG Pathway | hsa04110 | Cell cycle | 4 | -2.166291812 |
| KEGG Pathway | hsa05169 | Epstein-Barr virus infection | 5 | -2.14105423 |
| KEGG Pathway | hsa05203 | Viral carcinogenesis | 5 | -2.123523917 |

**Supplementary Table S2:** **Top enrichment function obtained by GO and KEGG analysis based on prognostic risk scores**
