# Supplementary material for: Identification of a necroptosis-related gene signature as a novel prognostic biomarker of cholangiocarcinoma
Source: Front Immunol. 2023 Mar 2;14:1118816. doi: 10.3389/fimmu.2023.1118816 (PMC10017743; doi:10.3389/fimmu.2023.1118816)
Supplement: Supplementary file 6 [file Table_3.docx]

**Supplementary Table S3: Top enrichment function obtained by GSEA analysis based on prognostic risk scores**

| Category | Description | NES | EnrichmentScore | Pvalue |
| --- | --- | --- | --- | --- |
| KEGG Pathway | Cytokine-cytokine receptor interaction | -1.602962639 | -0.418443143 | 0.001265823 |
| KEGG Pathway | Alcoholism | -1.67634025 | -0.457293939 | 0.001321004 |
| KEGG Pathway | Neutrophil extracellular trap formation | -1.546284609 | -0.421533233 | 0.001322751 |
| KEGG Pathway | Influenza A | -1.637194633 | -0.44942997 | 0.001335113 |
| KEGG Pathway | JAK-STAT signaling pathway | -1.86852967 | -0.515376109 | 0.001351351 |
| KEGG Pathway | Cell adhesion molecules | -1.830020828 | -0.509037174 | 0.001367989 |
| KEGG Pathway | Oxytocin signaling pathway | -1.607398085 | -0.447777623 | 0.001369863 |
| KEGG Pathway | Growth hormone synthesis, secretion and action | -1.533103451 | -0.439511864 | 0.001396648 |
| KEGG Pathway | Platelet activation | -1.613618369 | -0.46051787 | 0.001404494 |
| KEGG Pathway | Natural killer cell mediated cytotoxicity | -1.664558935 | -0.47505603 | 0.001404494 |
| KEGG Pathway | Osteoclast differentiation | -1.84938088 | -0.527964743 | 0.001412429 |
| KEGG Pathway | Relaxin signaling pathway | -1.614430408 | -0.460898774 | 0.00141844 |
| KEGG Pathway | Systemic lupus erythematosus | -1.751268638 | -0.4999643 | 0.00141844 |
| KEGG Pathway | Serotonergic synapse | -1.852526082 | -0.537705584 | 0.001428571 |
| KEGG Pathway | Th17 cell differentiation | -2.036497506 | -0.596061603 | 0.001440922 |
| KEGG Pathway | Leukocyte transendothelial migration | -1.554582773 | -0.451613933 | 0.001445087 |
| KEGG Pathway | Cholinergic synapse | -1.760558539 | -0.511873474 | 0.001445087 |
| KEGG Pathway | C-type lectin receptor signaling pathway | -1.612899677 | -0.474409 | 0.001455604 |
| KEGG Pathway | T cell receptor signaling pathway | -1.784352383 | -0.525439148 | 0.001461988 |
| KEGG Pathway | Protein digestion and absorption | -1.813452979 | -0.534008415 | 0.001461988 |
